# Testing patterns, patient and tumour characteristics and survival by *NRAS* and *KIT* genotype in melanoma

**DOI:** 10.1093/ced/llaf543

**Published:** 2025-12-11

**Authors:** Khaylen Mistry, Polly Jeffrey, Nick J Levell, Oliver Kennedy, Kathryn Richardson, Paul Craig, Chloe Bright, Siobhan Taylor, John Ragan, Dimitrios Karponis, Joanna Pethick, Katrina Lavelle, Fiona McRonald, Steven Hardy, Sally Vernon, Robert Dawe, Charlotte Proby, Paul Lorigan, Zoe C Venables

**Affiliations:** Department of Dermatology, Norfolk and Norwich University Hospitals Foundation Trust, Norwich, UK; Norwich Medical School, University of East Anglia, Norwich, UK; National Disease Registration Service, NHS England, Leeds, UK; National Disease Registration Service, NHS England, Leeds, UK; Department of Dermatology, Norfolk and Norwich University Hospitals Foundation Trust, Norwich, UK; Norwich Medical School, University of East Anglia, Norwich, UK; Department of Oncology, The Christie NHS Foundation Trust, Manchester, UK; University of Manchester, Manchester, UK; Norwich Epidemiology Centre, University of East Anglia, Norwich, UK; Departments of Cellular Pathology, North Bristol NHS Trust, Bristol, UK & Gloucestershire Hospitals NHS Foundation Trust, Cheltenham, UK; National Disease Registration Service, NHS England, Leeds, UK; Department of Cellular Pathology, Gloucestershire Hospitals NHS Foundation Trust, Cheltenham, UK; Patient and Public Representative, UK; Department of Dermatology, Norfolk and Norwich University Hospitals Foundation Trust, Norwich, UK; Norwich Medical School, University of East Anglia, Norwich, UK; National Disease Registration Service, NHS England, Leeds, UK; National Disease Registration Service, NHS England, Leeds, UK; National Disease Registration Service, NHS England, Leeds, UK; National Disease Registration Service, NHS England, Leeds, UK; National Disease Registration Service, NHS England, Leeds, UK; School of Medicine, University of Dundee, Dundee, UK; School of Medicine, University of Dundee, Dundee, UK; Department of Oncology, The Christie NHS Foundation Trust, Manchester, UK; University of Manchester, Manchester, UK; Department of Dermatology, Norfolk and Norwich University Hospitals Foundation Trust, Norwich, UK; Norwich Medical School, University of East Anglia, Norwich, UK; National Disease Registration Service, NHS England, Leeds, UK

## Abstract

**Background:**

*NRAS* and *KIT* mutations in melanoma bring implications for prognosis, follow-up, selection into trials and potential future treatment with targeted therapies. The frequency of *NRAS*/*KIT* mutations and their association with patient/tumour characteristics and survival is poorly documented.

**Objectives:**

To report national data from England on (i) the frequency of *NRAS* and *KIT* mutations, (ii) the association of patient/tumour characteristics with *NRAS* and *KIT* mutations, and (iii) the survival of patients with *NRAS* and *KIT* mutations.

**Methods:**

This retrospective cohort study identified all new melanomas diagnosed in England from 2016 to 2021 and molecular *NRAS*/*KIT* testing using data from the National Disease Registration Service. Multivariate logistic regression determined the association between (i) *NRAS* and *KIT* testing and patient/tumour characteristics, and (ii) *NRAS* genotype and patient/tumour characteristics. Age-standardized net survival (NS) analysed melanoma-specific mortality by *NRAS* and *KIT* genotype.

**Results:**

Of new melanomas diagnosed, 6.6% (6045/91 415) and 3.0% (2705/91 415) had an *NRAS* and *KIT* test registered. The proportion of successfully tested tumours that were *NRAS* and *KIT* mutated were 30.8% (1811/5887) and 5.8% (148/2560). East of England *NRAS* tested the highest proportion of cutaneous tumours (11.2%, 1114/9950) compared with the lowest in the North West (1.4%, 172/12 296). Older patients were more likely to have *NRAS* mutations [odds ratio (OR) 1.01, 95% confidence interval (CI) 1.01–1.02]. Women (OR 0.81, 95% CI 0.71–0.91) and those with head/neck melanomas (OR 0.37, 95% CI 0.31–0.44) were less likely to have *NRAS* mutations. There was no significant difference in 5-year NS between all-stage *NRAS* wildtype (WT) and mutated tumours (WT NS 62.3%, 95% CI 58.9–65.9 vs. mutated NS 58.5%, 95% CI 54.0–63.4). Similarly, there was no significant difference in 5-year NS between *KIT* WT and mutated tumours (WT NS 50.4%, 95% CI 44.7–57.3 vs. mutated NS 52.1%, 95% CI 37.1–73.2).

**Conclusions:**

This is the largest national dataset on melanoma *NRAS* and *KIT* status published to date to the best of our knowledge. Variations in *NRAS*/*KIT* testing by geographic/demographic factors drive initiatives to ensure consistent care. *NRAS*-mutated melanoma had a high incidence, which emphasizes the unmet need to develop therapies and trials for *NRAS*-mutated melanoma. This study increases our understanding of biomarkers *NRAS* and *KIT* and provides a foundation for optimizing melanoma care, contributing to advancements in precision oncology.

## Teaser

This retrospective cohort study is the largest national dataset on melanoma *NRAS* and *KIT* status ever published, to the best of our knowledge, with 6045 and 2705 tumours with an *NRAS* and *KIT* test registered in England. Regional and demographic variations in NRAS and *KIT* testing were identified, which drives initiatives to achieve optimal and consistent care. *NRAS*-mutated melanoma has a high incidence, underscoring the importance of future research to develop therapies for *NRAS*-mutated melanomas.

What is already known about this topic?

- *NRAS* and *KIT* mutations in melanoma have implications for prognosis, follow-up, selection into trials and the potential for future treatment with targeted therapies.
- In many countries internationally, there are no national guidelines for *NRAS* and *KIT* testing for melanoma.
- The frequency of *NRAS* and *KIT* mutations and their association with patient and tumour characteristics and survival are poorly documented with previous studies limited by small sample sizes.

What does this study add?

- This is the largest dataset on melanoma *NRAS* and *KIT* status published, to the best of our knowledge, with 6045 and 2705 tumours with an *NRAS* and *KIT* test registered, respectively.
- Variations in *NRAS* and *KIT* testing by geographic and demographic factors drive initiatives to achieve optimal and consistent care.
- *NRAS*-mutated melanoma has a high incidence, underscoring the importance of future research to develop targeted therapies for *NRAS*-mutated melanomas.

Mutations in the *BRAF*, *NRAS* and *KIT* genes are important oncogenic ‘drivers’ of melanoma. Since 2013, international guidance and expert consensus have recommended routinely determining *BRAF* status in stage III and IV melanoma, and some recommendations include stage II disease.^1–5^  *BRAF* mutations are present in approximately 30–50% of cutaneous melanomas, are more common in certain demographics such as younger patients and are prognostically significant and predictive of response to targeted therapy and immunotherapy in the adjuvant and palliative settings.^6–9^

By contrast, the frequency of *NRAS* and *KIT* mutations and their association with patient and tumour characteristics and survival are less well understood.^10,11^ Many previous epidemiology studies have been limited by small cohorts, with a lack of comprehensive data on a range of key covariates, and many studies pooled data for various cancers.^10^  *NRAS* and *KIT* mutations are important beyond their frequency because of their implications for prognosis, follow-up recommendations, selection into clinical trials and the unmet need for novel targeted therapies.

In the UK, there are no national guidelines for *NRAS*/*KIT* testing in specific melanoma subtypes. Current European guidelines recommend testing for *NRAS* and *KIT* mutations if the tumour is *BRAF* wildtype (WT) at the V600 locus.^4^ Many UK pathology laboratories do not routinely test for *NRAS*/*KIT* mutations, as these are not actionable mutations and most are selective about which melanoma types and stages they will request testing on. Sometimes *NRAS* testing is requested for more advanced stage melanoma and *KIT* testing for mucosal or acral lentiginous melanoma. The National Genomic Test Directory for Cancer in England was first published in 2018; its implementation drives access for all patients to next-generation sequencing panels that may provide greater information on the incidence of *NRAS* and *KIT* mutations.^12^

No national melanoma *NRAS* and *KIT* genetics data from England have been reported, to the best of our knowledge. The National Institute for Health and Care Excellence has recommended national cohort studies to evaluate melanoma biomarkers in treatment planning, response to treatment and prognosis.^1^ The aim of this study was to report national data for diagnoses of melanoma from England between 2016 and 2021 on (i) the frequency of molecular *NRAS* and *KIT* mutations, (ii) the association of patient demographics and tumour characteristics with *NRAS* and *KIT* mutations and (iii) the survival of patients with *NRAS* and *KIT* mutations.

## Patients and methods

### Study design, data sources and variables

This national cohort study used English cancer registry data from the National Disease Registration Service (NDRS).^13^ The registry maintains details of all cancers diagnosed each year across England (population 56 489 800 in the 2021 census).^14^ It is mandatory for all National Health Service (NHS) pathology laboratories, and recommended for all private pathology laboratories in England to provide all cancer pathology reports to the dataset. Only a small proportion are managed in the private sector in England and NDRS data are considered the gold-standard dataset for cancer data representation in England. Pathology report data are combined with the Patient Administration System and Cancer Outcomes and Services Dataset to form the National Cancer Registration Dataset (NCRD).^14^ The NDRS receives and records data on molecular genetic testing (including *NRAS* and *KIT* mutational analysis) directly from genomics or molecular pathology laboratories across England.

Patient records within the NCRD were linked to other datasets, including the National Radiotherapy Dataset, the Systemic Anti-Cancer Therapy (SACT) dataset, the NHS Hospital Episode Statistics datasets and death registrations from the Office for National Statistics.^15–17^ These linked data sources provided information on genetics and treatment pathways, including systemic therapy, radiotherapy and operation code data.

Data were extracted on 1 February 2024 for new melanomas diagnosed from 1 January 2016 to 31 December 2021, which was the latest available complete year. Melanoma was identified using International Classification of Diseases (ICD)-10 site codes and ICD-O-3 morphology and behaviour codes (Table S1; see Supporting Information).^18^ Disease-specific death was defined by the ICD-10 code C43 or C80 (melanoma skin cancer, malignant neoplasm without specification of site). Cause of death and vital status of patients was determined until 5 July 2023.

Patient variables extracted included age at diagnosis, gender, ethnicity, tumour site, stage at diagnosis, geography and deprivation. Table S2 (see Supporting Information) describes how variables were defined. Owing to low numbers for analysis, ethnicity was grouped into White and a minority ethnic grouping including Black, Asian, Chinese, Mixed and Other as per the Office for National Statistics categorization (see Table S2 for further information). REporting of studies Conducted using Observational Routinely-collected Data (RECORD) guidelines were adhered to (Appendix S1; see Supporting Information).

### Outcomes and statistical analysis

Data were extracted using Oracle SQL developer 19.4.0.354.1759. Statistical analyses were completed using R 4.3.2 and Stata 18.0. Multivariate logistic regression examined the association between *NRAS* and *KIT* testing of melanoma and covariates to understand any potential selection bias.

Multivariate logistic regression was used to examine the association between *NRAS* and *KIT* genotypes with covariates. The variance inflation factor was checked to ensure minimal multicollinearity. Secondary logistic regression evaluated whether tumours that were *NRAS* tested > 90 days from the pathological diagnosis date were more likely to be *NRAS* mutated, which may have suggested that patients with recurrent disease were more likely to be positive.

Survival time was calculated for patients diagnosed with their first malignant melanoma in England between 2016 and 2020. Patients were censored at the first occurrence of death, loss to follow-up (loss to NHS through lack of updated information or emigration) or the study end date (5 July 2023). The Pohar Perme estimator was used to calculate 5-year age-standardized net survival (NS) estimates by comparing the survival of patients with melanoma with that expected based on the general population of the same profile of age (single year), gender, deprivation and geographic region.^19–22^ Hazard ratios (HR) for disease-specific mortality were estimated using univariate and multivariate cause-­specific Cox regression models, with a second multivariate model including SACT limited to stage III/IV melanomas, with time to melanoma-specific death as the outcome. The proportional hazards assumption was examined for each covariate using log–log plots.

Sensitivity analysis to evaluate potential immortal time bias compared the HRs from the multivariate Cox models starting from the diagnosis date and *NRAS*/*KIT* testing date (cohort restricted to pathological diagnosis date – *NRAS*/*KIT* test date ≤ 90 days).

## Results

### *NRAS* testing patterns

Of new melanomas diagnosed, 6.6% (6045/91 415) had an *NRAS* test registered and 2.6% (158/6045) of those tested had either a failed, inconclusive or unknown test result. Of the successfully tested tumours, 95.7% (5634/5887) were cutaneous. The proportion of successfully tested tumours across all sites and cutaneous sites that were *NRAS* mutated was 30.8% (1811/5887) and 31.1% (1754/5634), respectively.

The percentage of stage III or IV cutaneous tumours that were *NRAS* tested was 22.4% (1958/8731), whereas the percentage of stage II and I tumours tested was 11.9% (2001/16 844) and 1.1% (574/52 353), respectively (Table S3; see Supporting Information). The East of England tested the highest proportion of cutaneous tumours, 11.2% (1114/9950), compared with the lowest in the North West, 1.4% (172/12 296).

Women [odds ratio (OR) 0.81, 95% confidence interval (CI) 0.76–0.86] were less likely to be *NRAS* tested. There was no association between age and *NRAS* testing. Mucosal melanomas (OR 2.08, 95% CI 1.73–2.49), melanomas at overlapping/unknown/external genital sites (OR 1.80, 95% CI 1.61–2.01), patients in the minority ethnic grouping (OR 1.31, 95% CI 1.06–1.62) and more deprived individuals (OR 1.12, 95% CI 1.01–1.25) were more likely to be *NRAS* tested.

### Patient and tumour characteristics and treatment received by *NRAS* genotype

In those tested, the study found there was a lower odds of having an *NRAS* mutation associated with patients who were women (OR 0.81, 95% CI 0.71–0.91) (Table 1). The *NRAS*-mutated genotype was associated with older age (OR 1.01, 95% CI 1.01–1.02). For cutaneous melanoma, the head/neck had the lowest odds of being *NRAS* mutated compared with the reference site of the limbs (OR 0.37, 95% CI 0.31–0.44) (Table 1). There was no association between whether a tumour was *NRAS* tested after 90 days from pathological diagnosis and *NRAS* genotype (OR 1.02, 95% CI 0.90–1.16) (Table S4; see Supporting Information).

**Table 1:** Odds ratios (ORs) for the association between covariates and mutated *NRAS* genotype

Variable	WT (*n* = 3880, 68.9%)	Mutated (*n* = 1754, 31.1%)	Total (*n* = 5634)	Logistic regression, OR^a^ (95% CI) (*n* = 5634)	
				Univariate analysis	Multivariate analysis
Gender					
Men	2290 (59.0)	1059 (60.4)	3349 (59.4)	REF	REF
Women	1590 (41.0)	695 (39.6)	2285 (40.6)	0.95 (0.84–1.06)	**0.81 (0.71–0.91)**
Age (years), median (IQR)	70 (48–92)	72 (54–90)	71 (50–92)	**1.01 (1.01–1.02)**	**1.02 (1.01–1.02)**
Site^b^					
Skin	3880 (95.2)	1754 (96.9)	5634 (95.7)	REF	REF
Mucosal	142 (3.5)	52 (2.9)	194 (3.3)	0.79 (0.56–1.09)	0.81 (0.57–1.13)
Other	54 (1.3)	5 (0.3)	59 (1.0)	**0.28 (0.11–0.57)**	**0.29 (0.12–0.61)**
Skin site					
Head/neck	842 (21.7)	226 (12.9)	1068 (19.0)	**0.44 (0.37–0.52)**	**0.37 (0.31–0.44)**
Limbs	1468 (37.8)	893 (50.9)	2361 (41.9)	REF	REF
Trunk	1100 (28.4)	445 (25.4)	1545 (27.4)	**0.67 (0.58–0.76)**	**0.64 (0.56–0.74)**
Overlapping/unknown/external genitals	470 (12.1)	190 (10.8)	660 (11.7)	**0.66 (0.55–0.80)**	**0.67 (0.55–0.83)**
Ethnicity^c^					
White	3607 (93.0)	1641 (93.6)	5248 (93.1)	REF	REF
Minority ethnic grouping	93 (2.4)	29 (1.7)	122 (2.2)	0.69 (0.44–1.03)	0.74 (0.47–1.13)
Black	13 (0.3)	4 (0.2)	17 (0.3)	–	**–**
Asian	19 (0.5)	8 (0.5)	27 (0.5)	–	**–**
Mixed	7 (0.2)	2 (0.1)	9 (0.2)	–	**–**
Other	54 (1.4)	15 (0.9)	69 (1.2)		
Unknown	180 (4.6)	84 (4.8)	264 (4.7)	1.03 (0.78–1.33)	1.07 (0.81–1.40)
Deprivation quintile					
1 (most deprived)	397 (10.2)	157 (9.0)	554 (9.8)	0.82 (0.66–1.01)	0.85 (0.68–1.06)
2	583 (15.0)	262 (14.9)	845 (15.0)	0.93 (0.77–1.11)	0.93 (0.77–1.12)
3	927 (23.9)	391 (22.3)	1318 (23.4)	0.87 (0.74–1.02)	0.85 (0.72–1.00)
4	946 (24.4)	447 (25.5)	1393 (24.7)	0.98 (0.84–1.14)	0.96 (0.82–1.13)
5 (least deprived)	1027 (26.5)	497 (28.3)	1524 (27.1)	REF	REF
Stage					
I	412 (10.6)	162 (9.2)	574 (10.2)	**0.77 (0.63–0.94)**	0.81 (0.66–1.00)
IA	98 (2.5)	33 (1.9)	131 (2.3)	–	–
IB	309 (8.0)	122 (7.0)	431 (7.6)	–	–
IX (stage I but not specified if IA/B)	5 (0.1)	7 (0.4)	12 (0.2)	–	–
II	1325 (34.1)	676 (38.5)	2001 (35.5)	REF	REF
IIA	271 (7.0)	178 (10.1)	449 (8.0)	–	–
IIB	464 (12.0)	269 (15.3)	733 (13.0)	–	–
IIC	584 (15.1)	228 (13.0)	812 (14.4)	–	–
IIX (stage II but not specified if IIA/B/C)	6 (0.2)	<5	7 (0.1)	–	–
III	986 (25.4)	411 (23.4)	1397 (24.8)	**0.82 (0.70–0.95)**	**0.83 (0.71–0.97)**
IIIA	123 (3.2)	28 (1.6)	151 (2.7)	–	–
IIIB	194 (5.0)	94 (5.4)	288 (5.1)	–	–
IIIC	408 (10.5)	186 (10.6)	594 (10.5)	–	–
IIID	39 (1.0)	8 (0.5)	47 (0.8)	–	–
IIIX (stage III but not specified if IIIA/B/C/D)	222 (5.7)	95 (5.4)	317 (5.6)	–	–
IV	394 (10.2)	167 (9.5)	561 (10.0)	0.83 (0.68–1.02)	0.87 (0.70–1.08)
IVX	394 (10.2)	167 (9.5)	561 (10.0)	–	–
Unknown	763 (19.7)	338 (19.3)	1101 (19.5)	0.87 (0.74–1.02)	0.90 (0.76–1.08)
Region					
London	433 (11.2)	195 (11.1)	628 (11.1)	0.98 (0.80–1.19)	1.03 (0.84–1.27)
East of England	771 (19.9)	343 (19.6)	1114 (19.8)	0.97 (0.82–1.14)	1.01 (0.85–1.20)
North East	292 (7.5)	121 (6.9)	413 (7.3)	0.90 (0.71–1.14)	0.98 (0.77–1.25)
North West	114 (2.9)	58 (3.3)	172 (3.1)	1.10 (0.79–1.53)	1.23 (0.87–1.72)
Yorkshire and the Humber	328 (8.5)	135 (7.7)	463 (8.2)	0.89 (0.71–1.12)	0.99 (0.84–1.94)
East Midlands	70 (1.8)	37 (2.1)	107 (1.9)	1.15 (0.75–1.72)	1.28 (0.84–1.94)
West Midlands	244 (6.3)	131 (7.5)	375 (6.7)	1.16 (0.92–1.48)	**1.32 (1.03–1.69)**
South East	1063 (27.4)	490 (27.9)	1553 (27.6)	REF	REF
South West	565 (14.6)	244 (13.9)	809 (14.4)	0.94 (0.78–1.13)	0.96 (0.79–1.16)

Data are *n* (%) unless otherwise specified. Data in bold are significant at *P* < 0.05 level. CI, confidence interval; REF, reference; WT, wildtype. ^a^ORs are for *NRAS* mutated (reference *NRAS* WT). Multivariate model includes gender, age, ethnicity, site, deprivation, stage, geographic region. Noncutaneous tumours were excluded from the multivariate model because of low counts of other/mucosal. A separate multivariate model with noncutaneous tumours was analysed for completeness. ^b^For site *n* = 5887 as includes noncutaneous tumours, with WT *n* = 4076 and mutant *n* = 1811. ^c^Please see Table S2 for further information.

Among patients with an *NRAS* mutation, 47.4% (805/1697) received immunotherapy only, 0.7% (12/1697) received targeted therapy only, 0.5% (9/1697) received both immunotherapy and targeted therapy and 51.3% (871/1697) received neither (Table S5; see Supporting Information).

### Survival by *NRAS* genotype

There was no significant difference in 5-year NS between people with all-stage *NRAS* WT and those with mutated tumours (WT NS 62.3%, 95% CI 58.9–65.9 vs. mutated NS 58.5%, 95% CI 54.0–63.4) (Table 2). Stage I *NRAS*-mutated genotype was associated with a lower 5-year NS (WT NS 70.0%, 95% CI 59.2–82.8 vs. mutated NS 27.9%, 95% CI 19.7–39.6).

**Table 2:** Five-year net survival (NS) by *NRAS* and *KIT* genotype

Stage, genotype, gender and age status	Total	5-year NS^a^ (95% CI)
*NRAS*	4050	
All stages *NRAS* WT	2772	62.3 (58.9–65.9)
All stages *NRAS* mutated	1278	58.5 (54.0–63.4)
Stage I *NRAS* WT	286	70.0 (59.2–82.8)
Stage II *NRAS* mutated	112	27.9 (19.7–39.6)
Stage II *NRAS* WT	1038	64.1 (58.7–70.0)
Stage II *NRAS* mutated	552	68.3 (62.1–75.1)
Stage IIA *NRAS* WT	223	71.3 (61.2–82.9)
Stage IIA *NRAS* mutated	157	64.1 (52.3–78.7)
Stage IIB *NRAS* WT	360	63.4 (53.3–75.5)
Stage IIB *NRAS* mutated	214	74.0 (64.2–85.3)
Stage IIC *NRAS* WT	453	58.8 (50.8–68.0)
Stage IIC *NRAS* mutated	180	57.1 (45.8–71.2)
Stage III *NRAS* WT	762	62.0 (55.6–69.2)
Stage III *NRAS* mutated	309	54.2 (45.6–64.5)
Stage IV *NRAS* WT	302	33.2 (26.3–42.0)
Stage IV *NRAS* mutated	120	33.5 (24.2–46.3)
Stage unknown *NRAS* WT	384	71.5 (62.9–81.3)
Stage unknown *NRAS* mutated	185	48.5 (34.3–68.5)
*KIT*	1482	
All stages *KIT* WT	1417	50.4 (44.4–57.3)
All stages *KIT* mutated	65	52.1 (37.1–73.2)

CI, confidence interval; WT, wildtype. ^a^Age-standardized NS at 1, 3 and 5 years was calculated by comparing overall survival from diagnosis date in patients with *NRAS*/*KIT* tested cutaneous melanoma between 2016 and 2020 compared with an age-, year-, gender-, deprivation- and geography-matched general population lifetable.

Similarly, in the multivariate Cox model, the *NRAS*-mutated genotype was not associated with a significant difference in disease-specific mortality (HR 1.12, 95% CI 1.00–1.25) (Table S6; see Supporting Information). SACT was not associated with disease-specific mortality in melanomas that underwent *NRAS* testing (HR 1.05, 95% CI 0.88–1.26) (Table S7; see Supporting Information). HRs from multivariate Cox models measuring survival from the diagnosis date or the *NRAS* testing date were similar, confirming minimal immortal time bias (HR 1.10, 95% CI 0.94–1.29) (Table S8; see Supporting Information).

### *KIT* testing patterns, characteristics and survival

Table 3 details the patient and tumour characteristics by *KIT* genotype. Of new melanomas diagnosed, 3.0% (2705/91 415) had a *KIT* test registered, of which 5.4% (145/2705) had either a failed, inconclusive or unknown test result. Of the successfully tested tumours, 85.1% (2178/2560) were cutaneous and 12.7% (326/2560) were mucosal (including external genital). The proportion of successfully tested tumours across all sites, cutaneous sites and mucosal sites (including external genital) that were *KIT* mutated was 5.8% (148/2560), 4.4% (95/2178) and 15.3% (50/326), respectively. The percentage of stage III or IV cutaneous tumours that were *KIT* tested was 9.2% (797/8640), whereas the percentage of stage II and I tumours tested was 4.4% (741/16 692) and 0.4% (184/52 289), respectively (Table S9; see Supporting Information).

**Table 3:** Patient and tumour characteristics by *KIT* genotype

Variables	Wildtype (*n* = 2083, 95.6%)	Mutated (*n* = 95, 4.4%)	Total (*n* = 2178)
Gender			
Men	1243 (59.7)	53 (56)	1296 (59.5)
Women	840 (40.3)	42 (44)	882 (40.5)
Age (years), median (IQR)	70 (49–91)	75 (60–90)	70 (49–91)
Site^a^			
Skin	2083 (86.4)	95 (64)	2178 (85.1)
Mucosal	276 (11.4)	50 (34)	326 (12.7)
Other	53 (2.2)	< 5	56 (2.2)
Skin site			
Head/neck	381 (18.3)	26 (27)	407 (18.7)
Limbs	919 (44.1)	46 (48)	965 (44.3)
Trunk	535 (25.7)	14 (15)	549 (25.2)
Overlapping/unknown	248 (11.9)	9 (9)	257 (11.8)
Ethnicity^b^			
White	1901 (91.3)	84 (88)	1985 (91.1)
Minority ethnic grouping	68 (3.3)	7 (7)	75 (3.4)
Unknown	114 (5.5)	< 5	118 (5.4)
Deprivation quintile			
1 (most deprived)	193 (9.3)	10 (11)	203 (9.3)
2	310 (14.9)	15 (16)	325 (14.9)
3	517 (24.8)	24 (25)	541 (24.8)
4	509 (24.4)	27 (28)	536 (24.6)
5 (least deprived)	554 (26.6)	19 (20)	573 (26.3)
Stage			
I	179 (8.6)	5 (5)	184 (8.4)
II	702 (33.7)	39 (41)	741 (34.0)
III	529 (25.4)	22 (23)	551 (25.3)
IV	235 (11.3)	11 (12)	246 (11.3)
Unknown	438 (21.0)	18 (19)	456 (20.9)
Region			
London	365 (17.5)	20 (21)	385 (17.7)
East of England	514 (24.7)	22 (23)	536 (24.6)
North East	29 (1.4)	< 5	29 (1.3)
North West	57 (2.7)	5 (5)	62 (2.8)
Yorkshire and the Humber	88 (4.2)	6 (6)	94 (4.3)
East Midlands	37 (1.8)	< 5	37 (1.7)
West Midlands	229 (11.0)	8 (8)	237 (10.9)
South East	352 (16.9)	19 (20)	371 (17.0)
South West	412 (19.8)	15 (16)	427 (19.6)
Management			
Definitive surgery	1806 (86.7)	77 (81)	1883 (86.5)
Systemic therapy	830 (39.8)	34 (36)	864 (39.7)
Radiotherapy	196 (9.4)	10 (11)	206 (9.5)
Systemic therapy^c^			
Immunotherapy only	815 (40.2)	36 (39)	851 (40.2)
Targeted therapy only	168 (8.3)	< 5	170 (8.0)
Targeted and immunotherapy	122 (6.0)	5 (5)	127 (6.0)
Neither	920 (45.4)	49 (53)	969 (45.8)

Data are *n* (%) unless otherwise specified. ^a^For site *n* = 2560 as includes noncutaneous tumours, with wildtype *n* = 2412 and mutant *n* = 148. ^b^Please see Table S2 for further information. ^c^For systemic therapy *n* = 2117 as counted at a patient level, with wildtype *n* = 2025 and mutant *n* = 92.

London tested the highest proportion of cutaneous tumours (5.4%, 385/7175) compared with the lowest in the North West (0.5%, 62/12 253). Patients who were women (OR 0.83, 95% CI 0.76–0.92) were less likely to be *KIT* tested. Mucosal (OR 8.04, 95% CI 5.84–11.03), cutaneous melanomas at overlapping or unknown sites (OR 1.61, 95% CI 1.36–1.90) and patients in the minority ethnic grouping (OR 1.78, 95% CI 1.37–2.30) were more likely to be KIT tested. A similar proportion of women who were *KIT* tested were *KIT* mutant compared with men [4.8% (42/882) vs. 4.1% (53/1296)].

There was no significant difference in 5-year NS between people with *KIT* WT and those with mutated tumours (*KIT* WT 5-year NS 50.4%, 95% CI 44.7–57.3 vs. *KIT*-mutated 5-year NS 52.1%, 95% CI 37.1–73.2) (Table 2).

## Discussion

This study presents the largest dataset on national melanoma *NRAS* and *KIT* genotype status published to date and the most complete reporting on testing patterns, characteristics and survival associated with *NRAS* and *KIT* genotypes, to the best of our knowledge. High-quality research has identified the need for detection of *BRAF*, *NRAS* and *KIT* mutations for metastatic or advanced acral and nail apparatus melanoma to provide the best therapeutic approach for patients.^23,24^

Marked geographical variation in *NRAS* and *KIT* testing across regions of England was observed, which may reflect the lack of national guidance for testing during the study period. Other explanations include regional differences in population characteristics, selective testing and variation in compliance in sending data between regional laboratories. Geographical testing patterns appeared similar for *NRAS* and *KIT*; therefore, variations in mutation testing methods (specific *NRAS* tests vs. panel testing with *BRAF*/*NRAS*/*KIT*) may have influenced results. Both *NRAS* and *KIT* testing were more common in patients who were men, had advanced melanoma, had mucosal melanoma, had melanoma in overlapping/unknown/external genital cutaneous sites and were in the minority ethnic grouping. Male patients and minority ethnic groups have been found to present with later stage melanoma, which may explain the higher testing.^10^  *KIT* mutations are associated with mucosal and acral melanoma and the greater testing in minority ethnic groups may be explained by the higher proportion of acral melanoma; however, no data were available for melanoma subtype.^10,25,26^

*NRAS* mutations were identified in 31.1% (1754/5634) of cutaneous tumours tested, almost double that of a recent global meta-analysis that reported 16.4% (641/3904). This discrepancy may reflect selection bias towards higher-risk tumours being tested.^10^ Another recent NDRS publication reported 35.2% (4401/12 490) of cutaneous tumours tested were *BRAF* mutated, which is lower than that routinely reported in the literature.^9^ These findings that *NRAS*-mutated (31.1%) and *BRAF*-mutated (35.2%) melanomas have a broadly similar incidence in melanomas undergoing testing in clinical practice are essential for clinical trial development as well as updating medical literature.^9^ As both mutations activate mitogen-activated protein kinase signalling, their mutual exclusivity was expected and confirmed; 97.3% (1745/1794) of *NRAS*-mutated tumours were *BRAF* WT.

*NRAS* mutations for all-stage melanoma were not significantly associated with survival. This is the case despite the absence of *NRAS*-directed therapies, in contrast with *BRAF*. The lower survival observed in stage I *NRAS*-mutated melanoma should be interpreted with caution given possible selective testing of ulcerated stage IB disease, the small sample size of stage I *NRAS*-tested melanoma and potential *NRAS* testing on recurrence. This dataset only recorded tumour stage at diagnosis and there was no validated marker for recurrence; hence, molecular testing may have occurred on disease recurrence. However, no association was found between a longer time lag between diagnosis date and genetic test date and *NRAS* genotype. Previous literature has associated *NRAS*-mutated melanoma with a higher tumour mutation burden and greater risk of brain metastases.^27,28^  *NRAS* mutations may be associated with a modest response to mitogen-activated protein kinase inhibitors, but the lack of specific *NRAS*-targeted therapies is an area of unmet need, particularly for advanced melanoma that has not responded to immunotherapy.^29–31^ A recent national qualitative study highlighted the development of effective *NRAS*-targeted therapies as a key priority for patients.^32^

*NRAS*-mutated genotype was associated with older adults, cutaneous sites and the limbs, in keeping with current literature.^10,33^ Growing evidence suggests *NRAS* mutations may be linked with chronic ultraviolet exposure, which may explain these observations.^34,35^ This study found men were more likely to have *NRAS* mutations, which is less well reported.^10^ In keeping with several meta-analyses, *KIT* mutations were more prevalent in mucosal melanomas.^10,36^ Survival analysis suggested *KIT* mutations did not have an impact on survival; however, analysis was limited by lack of follow-up time and sample size. Several phase II trials have observed modest response rates from tyrosine kinase inhibitors in *KIT*-mutated melanomas; however, the benefit was short lived.^37–39^ A key area of ongoing future research is to identify targeted therapies for *KIT*-mutated melanomas, particularly mucosal melanomas.^40^ Efforts to develop novel targeted therapies in *NRAS-*/*KIT*-mutated melanomas will hopefully deliver actionable biomarkers in addition to *BRAF*, reinforcing the importance of *NRAS*/*KIT* testing in high-risk melanomas.

Immunotherapy may yield a therapeutic avenue in *NRAS*- and *KIT*-mutated melanoma. A recent meta-­analysis of 1770 patients from 10 studies concluded *NRAS*-mutated melanoma demonstrated an increased likelihood of response to immunotherapy compared with *NRAS* WT.^41^ Further research exploring the effectiveness of immunotherapy in *KIT*-mutated melanoma is required; however, early signals suggest immunotherapy could offer promise in the treatment of *KIT*-mutated melanoma.^42^ Although this is encouraging, immunotherapy carries significant toxicities and may be contraindicated or poorly tolerated, endorsing the need for novel targeted therapies for *NRAS*-/*KIT*-mutated melanomas.

The main strength of this study is the overall size and quality of the data; however, because of low counts, analysis was limited to descriptive statistics for characteristics by *KIT* genotype. Additionally, regional differences in the completeness of molecular data submissions may have confounded regional comparisons. The study highlighted that factors such as gender, site, stage and geographical region influenced whether a melanoma was *NRAS* or *KIT* tested. As characteristics and survival by genotype were explored in the molecular tested cohort only, this cohort may not be representative of the wider melanoma population who did not undergo testing. This limited generalizability may apply in particular to patients with stage I/II disease who are less likely to be tested. As a result of the low numbers, analyses by ethnic group were restricted to White and a minority ethnic grouping (see Table S2). More diverse ethnicity data could be sourced from other national registries or more years of data.

In conclusion, by utilizing a national dataset, this study increases our understanding of melanoma biomarkers *NRAS* and *KIT*. Future endeavours should focus on addressing testing disparities and advancing targeted treatments to improve outcomes for patients with high-risk melanoma. Consistent mutational testing in advanced melanoma will prove imperative to support the development and deployment of targeted therapies. This evidence provides a strong foundation for optimizing melanoma care and contributing to global advancements in precision oncology.

## Supplementary Material

llaf543_Supplementary_Data

## Data Availability

The data underlying this article will be shared upon reasonable request to the corresponding author.
