# Supplementary material for: Testing patterns, patient and tumour characteristics and survival by NRAS and KIT genotype in melanoma
Source: Clin Exp Dermatol. 2025 Dec 11;51(4):604–11. doi: 10.1093/ced/llaf543 (PMC13020422; doi:10.1093/ced/llaf543)
Supplement: llaf543_Supplementary_Data [file llaf543_supplementary_data.zip › Supplementary tables.docx]

**Table S1 – classification of melanoma**

|  | ICD-10 site code | ICD-O-3 morphology code | ICD-O-3 behavior code |
| --- | --- | --- | --- |
| Melanoma | C43^a^, C510-512, C518, C519, C600-C602, C608, C609, C632, C000-C002, C006, C009 | 8720-8780^b^ | >2^c^ |

^a^ C43 = melanoma, C510 = labium majus, C511 = labium minus, C512 = clitoris, C518 = overlapping lesion of vulva, C519 = vulva unspecified, C600 = prepuce of penis, C601 = glans of penis, C602 = body of penis, C609 = penis unspecified, C632 = scrotum, C000 = external upper lip, C001 = external lower lip, C002 = external lip unspecified, C006 = commissure of lip, C009 = lip unspecified.

^b^ Morphology codes for naevi and melanomas

^c^ Behavior code >2 = 3 (malignant, primary site), 6 (malignant, metastatic site), 9 (malignant, uncertain site)

**Table S2 – Definitions of co-variates**

| Variate | Definition |
| --- | --- |
| Molecular genetics | Molecular genetics data included the name of the gene tested, date the gene was tested, whether the test was abnormal, mutation subtype and the time between the pathological diagnosis date and the test for NRAS/KIT status date. |
| Ethnicity* | Ethnicity data are recorded in secondary care from derived datasets and captured by the National Disease Registration Service (NDRS) from Cancer Outcomes and Services Dataset (COSD), Patient Administration System (PAS), the National Radiotherapy Dataset (RTDS) and Diagnostic Imaging Dataset (DID). If no ethnicity record can be extracted from the preceding data sources, an annual additional search is performed using Hospital Episode Statistics (HES) records. Where two conflicting ethnicity values are found, the NDRS record is updated with the most recently recorded known ethnicity. 18 ethnicity categories +1 ‘not stated’ category are defined by Office for National Statistics for the decennial census and used by NDRS. Ethnicity data in HES and census are self-reported. This systematic approach ensures ethnicity data is complete and reproducible. Ethnicity was grouped into white, grouped ethnic minoriies (Asian (excluding Chinese), Black, Chinese, mixed, other) and unknown. Due to low counts further categorisation of ethnicity beyond white and grouped ethnic minorities was not possible. Given the heterogenous nature of the ethnic minorities group findings should be interpreted with caution. |
| Skin site* | Skin site was grouped into head/neck (lip, eyelid, ear, face, scalp/neck), limbs (upper and lower), trunk, overlapping (multiple cutaneous sites)/unknown/external genitals. |
| All site** | All site data was grouped into skin (skin, lips, external genitals (scrotum, penile, vulva, labial, clitoris)), mucosal (nasal cavity, accessory sinuses, nasopharynx, oral, oesophageal, duodenum, rectal, anal, vaginal, cervical), ocular, other (lung, central nervous system, urinary tract, endocrine). |
| Deprivation | Deprivation quintiles were calculated using the Index of Multiple Deprivation equal Lower Super Output Area of residence (small geographical unit) weighted deprivation measures at the time of diagnosis. |
| Charlson co-morbidity score | Charlson co-morbidity score was defined by the summed cancer and non-cancer component from Hospital Episode Statistics (HES) data with a look back period of 3 to 27 months from cancer diagnosis date. |
| Geographical region | Geography was defined as the government office region of residence at the time of diagnosis. There are 9 regions in England. |
| Stage | Staging data reflected stage at diagnosis. The majority of tumors were staged in Union for International Cancer Control (UICC) 8 or (American Joint Committee on Cancer) AJCC 7, with a small number in earlier versions of UICC or AJCC. |
| Previous cancer | Previous cancer was defined using International Classification of Diseases (ICD)-10 site codes and ICD-O3 morphology and behavior codes as any cancer (excluding non-melanoma skin cancer) within five years prior to melanoma diagnosis. |
| Surgery | Surgical codes were retrieved from the HES and the National Cancer Registration Dataset (NCRD) > -30 days from the melanoma diagnosis. Definitive surgery included wide local excision, Moh’s micrographic surgery and other complex excisions such as flaps or grafts. |
| Radiotherapy | Radiotherapy (> -30 days from the melanoma diagnosis) data were derived from Radiotherapy Dataset (RTDS). |
| Systemic therapy | Systemic Anti-Cancer Therapy (SACT) (> -30 days from the melanoma diagnosis) data were derived from the SACT dataset. SACT encompassed immunotherapy and targeted therapy. Targeted therapy included BRAF inhibitors, MEK inhibitors and tyrosine kinase inhibitors. SACT regimens were combined so that a patient was counted once. SACT intent coding were not reported due to incompleteness and known inconsistencies in coding. |

* Grouping site and ethnicity data ensured sufficient counts to maintain anonymity and stabilise the multivariate models whilst maintaining an acceptable categorisation in line with current literature and clinical expertise.

**For NRAS and KIT, ocular and other grouped together to stabilise regression models. For KIT, external genital melanoma was grouped with mucosal melanoma rather than cutaneous melanoma

| **Variable** | **Not tested,**  **n = 81819 (93.6%)** | **NRAS tested ,**  **n = 5634**  **(6.4%)** | **Total,**  **n = 87453** | **Logistic regression, multivariable analysis, n = 87453, OR* (95% CI)** |
| --- | --- | --- | --- | --- |
| **Gender** |  |  |  |  |
| Male | 40807 (49.9) | 3349 (59.4) | 44156 (50.5) | REF |
| Female | 41012 (50.1) | 2285 (40.6) | 43297 (49.5) | **0.81 (0.76-0.86)** |
| **Age band** |  |  |  |  |
| <70 | 47031 (57.5) | 2637 (46.8) | 49668 (56.8) | REF |
| 70-79 | 19945 (24.4) | 1639 (29.1) | 21584 (24.7) | 1.06 (0.99-1.14) |
| >=80 | 14843 (18.1) | 1358 (24.1) | 16201 (18.5) | 0.96 (0.89-1.03) |
| **Site**** |  |  |  |  |
| Skin | 81819 (95.7) | 5634 (95.7) | 87453 (95.7) | REF |
| Mucosal | 549 (0.6) | 194 (3.3) | 739 (0.8) | **2.08 (1.73-2.49)** |
| Other | 3154 (3.7) | 59 (1.0) | 3223 (3.7) | **0.15 (0.11-0.19)** |
| **Skin site** |  |  |  |  |
| Head/neck | 14340 (17.5) | 1068 (19.0) | 15408 (17.6) | 1.01 (0.93-1.10) |
| Limbs | 38851 (47.4) | 2361 (41.9) | 41212 (47.1) | REF |
| Trunk | 26101 (31.9) | 1545 (27.4) | 27646 (31.6) | 0.93 (0.87-1.01) |
| Overlapping/unknown | 2527 (3.1) | 660 (11.7) | 3187 (3.6) | **1.80 (1.61-2.01)** |
| /external genitals |  |  |  |  |
| **Ethnicity** |  |  |  |  |
| White | 74901 (91.5) | 5248 (93.1) | 80149 (91.6) | REF |
| Grouped ethnic minorities | 945 (1.2) | 122 (2.2) | 1067 (1.2) | **1.31 (1.06-1.62)** |
| Unknown | 5973 (7.3) | 264 (4.7) | 6237 (7.1) | **0.75 (0.65-0.85)** |
| **Deprivation quintile** |  |  |  |  |
| 1 (most deprived) | 8696 (10.6) | 554 (9.8) | 9250 (10.6) | **1.12 (1.01-1.25)** |
| 2 | 12523 (15.3) | 845 (15.0) | 13368 (15.3) | 1.02 (0.93-1.12) |
| 3 | 17059 (20.8) | 1318 (23.4) | 18377 (21.0) | **1.16 (1.07-1.26)** |
| 4 | 20301 (24.8) | 1393 (24.7) | 21694 (24.8) | **1.10 (1.02-1.19)** |
| 5 (least deprived) | 23240 (28.4) | 1524 (27.1) | 24764 (28.3) | REF |
| **Stage** |  |  |  |  |
| 1 | 51779 (63.3) | 574 (10.2) | 52353 (59.9) | REF |
| 2 | 14843 (18.1) | 2001 (35.5) | 16844 (19.3) | **12.12 (11.01-13.36)** |
| 3 | 4929 (6.0) | 1397 (24.8) | 6326 (7.2) | **26.57 (23.96-29.49)** |
| 4 | 1844 (2.3) | 561 (10.0) | 2405 (2.8) | **22.61 (23.96-29.49** |
| Unknown | 8424 (10.3) | 1101 (19.5) | 9525 (10.9) | **9.95 (8.92-11.11)** |
| **Region** |  |  |  |  |
| London | 6577 (8.0) | 628 (11.1) | 7205 (8.2) | 0.93 (0.83-1.03) |
| East of England | 8836 (10.8) | 1114 (19.8) | 9950 (11.4) | **1.16 (1.06-1.27)** |
| North-East | 4852 (5.9) | 413 (7.3) | 5265 (6.0) | 1.02 (0.90-1.15) |
| North-West | 12124 (14.8) | 172 (3.1) | 12296 (14.1) | **0.12 (0.11-0.15)** |
| Yorkshire and the Humber | 7294 (8.9) | 463 (8.2) | 7760 (8.9) | **0.58 (0.51-0.65)** |
| East Midlands | 6958 (8.5) | 107 (1.9) | 7065 (8.1) | **0.13 (0.11-0.16)** |
| West Midlands | 7526 (9.2) | 375 (6.7) | 7901 (9.0) | **0.44 (0.39-0.49)** |
| South-East | 16095 (19.7) | 1553 (27.6) | 17648 (20.2) | REF |
| South-West | 11554 (14.1) | 809 (14.4) | 12363 (14.1) | **0.65 (0.51-0.65)** |

**Table S3 – Logistic regression of co-variates associated with NRAS testing**

*Odds ratio are for NRAS tested (reference not NRAS tested). Multivariate model includes gender, age, site, ethnicity, deprivation, stage, geographic region. Non-cutaneous tumours were excluded from the multivariate model due to low counts of other/mucosal. A separate multivariate model with non-cutaneous tumours included was analysed for completeness.

**for site n = 91415 (Not NRAS tested = 85528, NRAS tested = 5887)

**Table S4 - Logistic regression of the association of NRAS genotype with the time between the pathological diagnosis date and the test for NRAS status date (NRAS tested within 90 days of diagnosis)**

| **Variable** | **Wild type (WT), n = 3880 (68.9%)** | **Mutated, n = 1754 (31.1%)** | **Total, n = 5634** | **Logistic regression, multivariate analysis, n = 5634, OR* (95% CI)** |
| --- | --- | --- | --- | --- |
| **NRAS testing** |  |  |  |  |
| **Tested <90 days** | 2345 (60.4) | 1052 (60.0) | 3397 (60.3) | REF |
| **Tested** $\boldsymbol{\geq}$**90 days** | 1535 (39.6) | 702 (40.0) | 2237 (39.7) | 1.02 (0.90-1.16) |

*Odds ratio are for not NRAS tested within 90 days of diagnosis (reference tested within 90 days). Multivariate model includes age, gender, site, ethnicity, deprivation, geographic region and stage. Non-cutaneous tumours were excluded from the multivariate model due to low counts of other/mucosal.

**Table S5 – Treatment received by NRAS genotype**

| **Variable** | **Wild type (WT), n = 3880 (68.9%)** | **Mutated, n = 1754 (31.1%)** | **Total, n = 5634** |
| --- | --- | --- | --- |
| **Management** |  |  |  |
| Definitive surgery | 3413 (88.0) | 1575 (90.0) | 4988 (88.5) |
| Systemic therapy | 1392 (35.9) | 552 (31.5) | 1944 (34.5) |
| Radiotherapy | 320 (8.2) | 145 (8.3) | 465 (8.3) |
| **Systemic therapy*** |  |  |  |
| Immunotherapy only | 1026 (27.3) | 805 (47.4) | 1831 (33.5) |
| Targeted therapy only | 479 (12.8) | 12 (0.7) | 491 (9.0) |
| Targeted and immunotherapy | 337 (9.0) | 9 (0.5) | 346 (14.2) |
| Neither | 1913 (50.9) | 871 (51.3) | 2784 (51.1) |

*For systemic therapy n=5452 as counted at a patient level

**Table S6 – Univariate and multivariate Cox model for disease-specific death by NRAS genotype**

| **Variable** | **Total, n = 4050 (100%)** | **Melanoma-specific death, n = 1375 (100%)** | **Cox regression, univariate analysis, n = 4050, HR* (95% CI)** | **Cox regression, multivariate analysis, n = 4050, HR* (95% CI)** |
| --- | --- | --- | --- | --- |
| **NRAS genotype** |  |  |  |  |
| Wild-type (WT) | 2772 (68.4) | 911 (66.3) | REF | REF |
| Mutated | 1278 (31.6) | 464 (33.7) | 1.11 (0.90-1.25) | 1.12 (1.00-1.25) |

*Hazard ratio is for disease-specific death defined as C43/C80 measuring survival from diagnosis date. Multivariate model includes gender, age, site, ethnicity, deprivation, stage, geographic region, Charlson score, previous cancer in past five years, age^2^, age-gender interaction, age^2^-gender interaction. Non-cutaneous tumours were excluded from the multivariate model due to low counts of other/mucosal.

**Table S7 – Univariate and multivariate Cox model for disease-specific death by SACT for NRAS tested melanoma**

| **Variable** | **Total, n = 1493 (100%)** | **Melanoma-specific death, n = 614 (100%)** | **Cox regression, univariate analysis, n = 1493, HR* (95% CI)** | **Cox regression, multivariate analysis, n = 1493, HR* (95% CI)** |
| --- | --- | --- | --- | --- |
| **SACT** | 822 (55.1) | 351 (57.2) | **1.20 (1.02-1.42)** | 1.05 (0.88-1.26) |

*Hazard ratio is for disease-specific death defined as C43/C80 measuring survival from diagnosis date. Multivariate model includes gender, age, site, ethnicity, deprivation, stage, geographic region, Charlson score, previous cancer in past five years, age^2^, age-gender interaction, age^2^-gender interaction and SACT (immunotherapy and targeted therapy). Non-cutaneous tumours were excluded from the multivariate model due to low counts of other/mucosal. Stage was restricted to stage III/IV tumours (total (n = 1493), melanoma specific death (n = 614). SACT was not further divided into immunotherapy only, targeted only, both or neither due to low counts.

**Table S8 – Multivariate Cox model for disease-specific death by NRAS genotype where survival time starting from the diagnosis date and NRAS testing date**

| **Variable** | **Total, n = 2166 (100%)** | **Melanoma-specific death, n = 705 (100%)** | **Cox regression, multivariate analysis, measuring survival from genetic test date, n = 2166, HR* (95% CI)** | **Cox regression, multivariate analysis, measuring survival from diagnosis date, n = 2166, HR* (95% CI)** |
| --- | --- | --- | --- | --- |
| **NRAS genotype** |  |  |  |  |
| Wild-type (WT) | 1484 (68.5) | 474 (67.2) | REF | REF |
| Mutated | 682 (31.5) | 231 (32.8) | 1.10 (0.94-1.29) | 1.10 (0.94-1.29) |

*Hazard ratio is for disease-specific death defined as C43/C80. Multivariate model includes gender, age, site, ethnicity, deprivation, stage, geographic region, Charlson score. Non-cutaneous tumours were excluded from the multivariate model due to low counts of other/mucosal. Cohort restricted to pathological diagnosis date - test for NRAS status date ≤ 90 days.

**Table S9 – Logistic regression of co-variates associated with KIT testing**

| **Variable** | **Not tested, n = 84898 (93.6%)** | **KIT tested, n = 2178**  **(6.4%)** | **Total, n = 87076** | **Logistic regression, multivariable analysis, n = 87076, OR* (95% CI)** |
| --- | --- | --- | --- | --- |
| **Gender** |  |  |  |  |
| Male | 42816 (50.4) | 1296 (59.5) | 44112 (50.7) | REF |
| Female | 42082 (49.6) | 882 (40.5) | 42964(49.3) | **0.83 (0.76-0.92)** |
| **Age band** |  |  |  |  |
| <70 | 48452 (57.1) | 1041 (47.8) | 49493 (56.8) | REF |
| 70-79 | 20858 (24.6) | 626 (28.7) | 21484 (24.7) | 1.03 (0.93-1.15) |
| >=80 | 15588 (18.4) | 511 (23.5) | 16099 (18.5) | 0.91 (0.81-1.02) |
| **Site**** |  |  |  |  |
| Skin | 84898 (95.5) | 2178 (85.1) | 87076 (95.2) | REF |
| Mucosal | 790 (0.9) | 326 (12.7) | 1116 (1.2) | **8.04 (5.84-7.83)** |
| Other | 3167 (3.6) | 56 (2.2) | 3223(3.5) | **0.34 (0.24-0.42)** |
| **Skin site** |  |  |  |  |
| Head/neck | 15001 (17.7) | 407 (18.7) | 15408 (17.7) | 0.95 (0.84-1.08) |
| Limbs | 40247 (47.4) | 965 (44.3) | 41212 (47.3) | REF |
| Trunk | 27097 (31.9) | 549 (25.2) | 27646 (31.7) | **0.81 (0.72-0.91)** |
| Overlapping/unknown | 2553 (3.0) | 257 (11.8) | 2810 (3.2) | **1.61 (1.36-1.90)** |
|  |  |  |  |  |
| **Ethnicity** |  |  |  |  |
| White | 77813 (91.6) | 1985 (91.1) | 79798 (91.6) | REF |
| Grouped ethnic minorities | 979 (1.2) | 75 (3.4) | 1054 (1.2) | **1.78 (1.37-2.30)** |
| Unknown | 6106 (7.2) | 118 (5.4) | 6224 (7.1) | 0.95 (0.78-1.15) |
| **Deprivation quintile** |  |  |  |  |
| 1 (most deprived) | 8984 (10.6) | 203 (9.3) | 9187 (10.6) | 1.12 (0.95-1.33) |
| 2 | 12977 (15.3) | 325 (14.9) | 13302 (15.3) | 0.94 (0.82-1.09) |
| 3 | 17758 (20.9) | 541 (24.8) | 18299 (21.0) | 1.13 (1.00-1.28) |
| 4 | 21078 (24.8) | 536 (24.6) | 21614 (24.8) | 1.05 (0.93-1.19) |
| 5 (least deprived) | 24101 (28.4) | 573 (26.3) | 24674 (28.3) | REF |
| **Stage** |  |  |  |  |
| 1 | 52105 (61.4) | 184 (8.4) | 52289 (60.0) | REF |
| 2 | 15951 (18.8) | 741 (34.0) | 16692 (19.2) | **12.83 (10.90-15.18)** |
| 3 | 5716 (6.7) | 551 (25.3) | 6267 (7.2) | **26.85 (22.67-31.95)** |
| 4 | 2127 (2.5) | 246 (11.3) | 2373 (2.7) | **26.17 (21.23-32.30)** |
| Unknown | 8999 (10.6) | 456 (20.9) | 9455 (10.9) | **11.39 (9.52-13.67)** |
| **Region** |  |  |  |  |
| London | 6790 (8.0) | 385 (17.7) | 7175 (8.2) | **2.55 (2.19-2.87)** |
| East of England | 9379 (10.8) | 536 (24.6) | 9915 (11.4) | **2.44 (2.12-2.80)** |
| North-East | 5210 (5.9) | 29 (1.3) | 5239 (6.0) | **0.29 (0.20-0.42)** |
| North-West | 12191 (14.8) | 62 (2.8) | 12253 (14.1) | **0.23 (0.17-0.30)** |
| Yorkshire and the Humber | 7625 (8.9) | 94 (8.2) | 7719 (8.9) | **0.53 (0.42-0.66)** |
| East Midlands | 6980 (8.5) | 37 (4.3) | 7017 (8.1) | **0.23 (0.16-0.32)** |
| West Midlands | 7607 (9.2) | 237 (10.9) | 7844 (9.0) | **1.36 (1.15-1.61)** |
| South-East | 17217 (19.7) | 371 (17.9) | 17588 (20.2) | REF |
| South-West | 11899 (14.1) | 427 (19.6) | 12326 (14.2) | **1.61 (1.39-1.87)** |

*Odds ratio are for KIT tested (reference not KIT tested). Multivariate model includes gender, age, site, ethnicity, deprivation, stage, geographic region. Non-cutaneous tumours were excluded from the multivariate model due to low counts of other/mucosal. A separate multivariate model with non-cutaneous tumours included was analysed for completeness. For site multivariate model excluded geographic region due to low counts.

**for site n = 91415 (Not KIT tested = 88855, KIT tested = 2560)
